# FungiDB: An Integrated Bioinformatic Resource for Fungi and Oomycetes

**DOI:** 10.3390/jof4010039

**Published:** 2018-03-20

**Authors:** Evelina Y. Basenko, Jane A. Pulman, Achchuthan Shanmugasundram, Omar S. Harb, Kathryn Crouch, David Starns, Susanne Warrenfeltz, Cristina Aurrecoechea, Christian J. Stoeckert, Jessica C. Kissinger, David S. Roos, Christiane Hertz-Fowler

**Affiliations:** 1 Centre for Genomic Research, Institute of Integrative Biology, University of Liverpool, Liverpool L69 7ZB, UK; Jane.Pulman@liverpool.ac.uk (J.A.P.); achchu@liverpool.ac.uk (A.S.); D.E.Starns@liverpool.ac.uk (D.S.); 2 Department of Biology, University of Pennsylvania, Philadelphia, PA 19104, USA; oharb@sas.upenn.edu (O.S.H.); droos@sas.upenn.edu (D.S.R.); 3 Wellcome Trust Centre for Molecular Parasitology, Glasgow G12 8TA, UK; kathryn.crouch@glasgow.ac.uk; 4 Center for Tropical and Emerging Global Diseases, Institute of Bioinformatics, University of Georgia, Athens, GA 30602, USA; swfeltz@uga.edu (S.W.); aurreco@uga.edu (C.A.); jkissing@uga.edu (J.C.K.); 5 Department of Genetics, University of Pennsylvania, Philadelphia, PA 19104, USA; stoeckrt@pennmedicine.upenn.edu

**Keywords:** fungi, pathogen, bioinformatics, omics, genomics, transcriptomics, proteomics, sequence analysis, RNA-Seq, Galaxy

## Abstract

FungiDB (fungidb.org) is a free online resource for data mining and functional genomics analysis for fungal and oomycete species. FungiDB is part of the Eukaryotic Pathogen Genomics Database Resource (EuPathDB, eupathdb.org) platform that integrates genomic, transcriptomic, proteomic, and phenotypic datasets, and other types of data for pathogenic and nonpathogenic, free-living and parasitic organisms. FungiDB is one of the largest EuPathDB databases containing nearly 100 genomes obtained from GenBank, *Aspergillus* Genome Database (AspGD), The Broad Institute, Joint Genome Institute (JGI), Ensembl, and other sources. FungiDB offers a user-friendly web interface with embedded bioinformatics tools that support custom in silico experiments that leverage FungiDB-integrated data. In addition, a Galaxy-based workspace enables users to generate custom pipelines for large-scale data analysis (e.g., RNA-Seq, variant calling, etc.). This review provides an introduction to the FungiDB resources and focuses on available features, tools, and queries and how they can be used to mine data across a diverse range of integrated FungiDB datasets and records.

## 1. Introduction

The Eukaryotic Pathogen Bioinformatics Resource Center (eupathdb.org) comprises a family of bioinformatics resources that include an integrated functional genomics database for fungi and oomycetes—FungiDB [1,2]. FungiDB (fungidb.org) is a free online resource for data mining and functional genomics analysis. It provides an easy-to-use, interactive interface to explore genomes, annotation, functional data (e.g., transcriptomics, proteomics, phenomic data for selected species), metabolic pathways, and results from numerous genome-wide analyses (i.e., InterPro scan, signal peptide and transmembrane domain predictions, orthology, etc.). FungiDB contains an expanding number of genomes from various taxa including, but not limited to, plant, animal, and human pathogens. FungiDB contains nearly 100 genomes obtained from GenBank [3], Ensembl [4], Joint Genome Institute (JGI) [5], *Aspergillus* Genome Database (AspGD) [6], *Candida* Genome Database (CGD) [7], *Saccharomyces* Genome Database (SGD) [8], PomBase [9], and The Broad Institute [10]. FungiDB continues to acquire new genomes and datasets from data repositories, other fungal databases, and individual providers. The loaded genomes and related datasets can be investigated via a number of search strategies and FungiDB website-embedded tools. Presented here is an overview of FungiDB resources and several tutorials on how to mine FungiDB bioinformatic resources by creating in silico experiments.

## 2. Overview of the FungiDB Home Page and its Features

**Figure 1:**
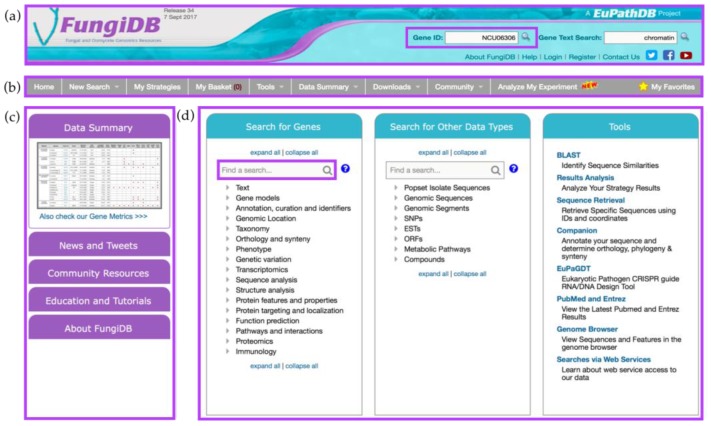
FungiDB Home page and its features. The home page comprises (**a**) the header section with *Gene ID* search highlighted in purple; (**b**) the main menu in grey; (**c**) the side bar with links and various information sections; and (**d**) the main component offering *Search for Genes*, *Search for Other Data Types*, and *Tools* sections. *Find a search* box from *Search for Genes* component is highlighted in purple.

The home page of FungiDB can be divided into four sections: the header (Figure 1a), the grey menu bar (Figure 1b), the side bar on the left (Figure 1c), and the main central section with three large panels: *Search for Genes* (left), *Search for Other Data Types* (middle), and *Tools* (right) (Figure 1d). 

The header section offers quick access to the *Gene ID* and *Gene Text search* and a list of useful links (Figure 1a). To quickly navigate to a specific gene record page, a user can enter a single Gene ID (e.g., NCU06306) into the *Gene ID* search box at the top of the main page (Figure 1a). Alternatively, searching for *chromatin* in the *Gene Text Search* box adjacent to the *Gene ID* search box will return a list of genes from fungal and oomycete organisms that have matches to the searched term in any of the following gene fields: enzyme commission (EC) description, Gene ID, Gene notes, gene product, gene name, gene ontology (GO) terms and definitions, metabolic pathway names and descriptions, phenotype, protein domain names and descriptions, PubMed, and user comments. The links located directly under the search boxes provide quick access to information about the FungiDB project, login and registration, social media links, YouTube tutorial channel, and the *Contact Us* email form. Support inquiries can also be sent to *help@fungidb.org* (Figure 1a).

The grey menu bar is preserved across almost all FungiDB pages and provides easy navigation to all database sections when a user navigates away from the main page (Figure 1b). The side bar section (Figure 1c) consists of the *Data Summary*, *News and Tweets*, *Community Resources*, *Education and Tutorials*, and *About FungiDB* sections.

The central section is the largest section of the home page and consists of three panels: *Search for Genes, Search for Other Data Types*, and *Tools* panels (Figure 1d). The *Search for Genes* panel contains links to searches organized into seventeen intuitive categories allowing the user to quickly navigate to a desired search. Each search initiated from this panel will return lists of genes as search results. For example, a search for genes based on a specific organism is found under the *Taxonomy* category. Secondly, the *Search for Other Data Types* panel offers searches that return non-gene entities (e.g., *Single Nucleotide Polymorphisms (SNPs)*, *Metabolic Pathways*, *Compounds*, *Genomic Sequences based on genomic location*). Non-gene entities that have genomic locations can be co-located with each other or with genes (see below). Both the *Search for Genes* and *Search for Other Data Types* panels include a find option that allows users to quickly find a specific search by typing a key word in the *Find a search* box (highlighted in purple). Lastly, the third panel on the right provides access to useful bioinformatics tools that can be used independently of the searches (e.g., BLAST, Sequence Retrieval Tool, or Genome Browser).

## 3. Exploring FungiDB Records: Gene ID Searches and Gene Record Pages

**Figure 2:**
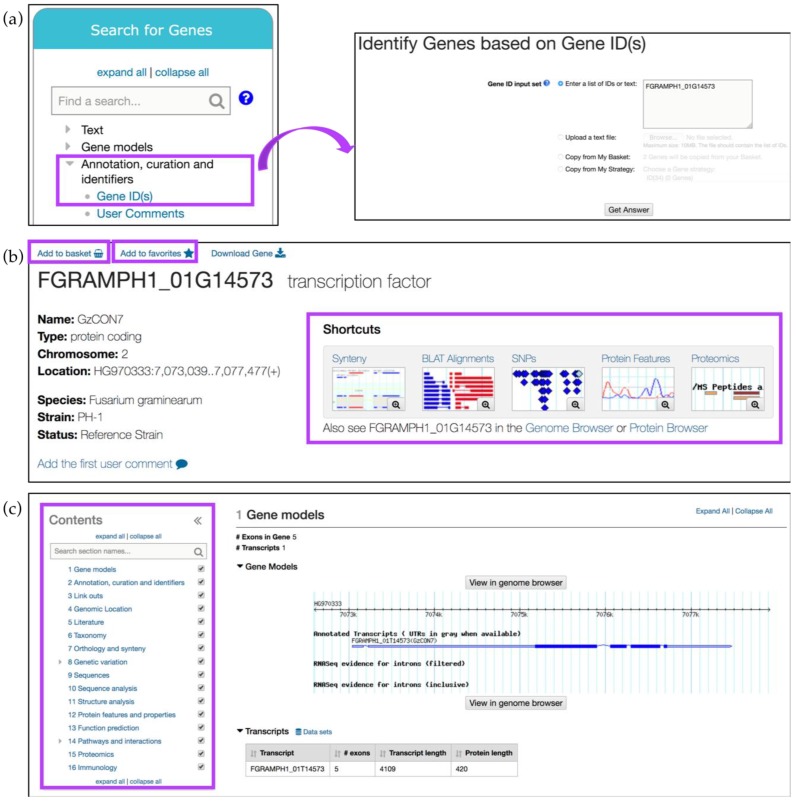
Simple searches and gene record pages. (**a**) A Gene ID search can be initiated from the main component section, *Search for Genes* option, *Annotation, curation and identifiers* submenu (highlighted in purple); (**b**) Gene record page can be saved or bookmarked via *Add to basket* or *Add to favorites* links, respectively (highlighted in purple). Thumbnail *Shortcuts* (highlighted) provides easy navigation to key datasets; (**c**) Additional gene record sections can be quickly identified via the searchable *Contents* menu on the left (highlighted in purple).

A *Gene ID* search can also be invoked from the *Search for Genes* panel via the *Annotation, curation, and identifiers* category (Figure 2a). For example, searching for FGRAMPH1_01G14573, a *Fusarium graminearum* (*F. graminearum*) transcription factor important for virulence [11], returns the gene record page for this gene. Gene record pages contain aggregate information about a gene and its function that is generated from integrated datasets and automated pipelines.

Gene record pages can be saved (via the *Add to basket* link) or bookmarked (by clicking on the *Add to favorites* link) (Figure 2b). The *Add to basket* function saves the gene record to a basket associated with a user’s account. Basket items can be found in the *My Strategies* section when a user is logged in. The basket serves as a shopping cart where genes in the basket can be downloaded or transferred to a search strategy. Adding a gene to favorites creates a bookmark to that gene in the *My Favorites* section available within the grey menu bar (Figure 1b). In the *My Favorites* section, users can also add private notes and project descriptions about saved items.

FungiDB gene record pages can be navigated via the thumbnail *Shortcuts* menu at the top (Figure 2b) and *Contents* menu on the left (Figure 2c). For example, when transcriptomics data for a gene of interest is integrated into FungiDB, a corresponding thumbnail appears on the gene page. Each thumbnail shows the number of datasets available within a given category. Clicking on the magnifying glass symbol within the thumbnail will open a preview screen of the evidence, while clicking anywhere within the thumbnail will navigate the user to the corresponding section on the gene page. Sections can be quickly identified by using the *Search section names* search box at the top of the *Contents* menu and clicking on the desired section. In addition, sections in a gene page can be hidden by unchecking the box to the right of any section name in the *Contents* menu.

The *Gene models* section is the first section of the gene record page and it contains information about the structure of the gene such as exon count, transcript number and a visual GBrowse representation of gene location, annotated UTRs and introns, and RNA-Seq evidence (when available). This section can be viewed in more detail, including the supporting data, by clicking on the *View in genome browser* button.

The *Annotation, curation, and identifiers* section offers alternate product descriptions, previous identifiers and aliases, notes from annotator, and user comments associated with a gene record. These sections are populated using data from either other fungal specialized resources, internal curation, or user-submitted data (user comments, see below).

The *Link outs* section offers redirection to other resources including EnsemblFungi [4], Entrez Gene [12], FGSC [13], UniProt [14], JGI [5], PHI-Base [15], AspGD [6], PhylomeDB [16], PomBase [9], CGD [7], SGD [8], and others.

The *Genomic Location* section contains gene or sequence coordinates within a chromosome or contig/scaffold and a link out to GBrowse.

The *Literature* section provides access to associated publications gathered by FungiDB via user comments or direct uploads from other resources (The Broad Institute, AspGD, fgmutantDB [17], PHI-Base, The *Neurospora crassa* e-compendium [18]) and FungiDB in-house curation efforts.

The *Taxonomy* section provides detailed information about organism classification within the Eukaryote kingdom following the National Center for Biotechnology Information (NCBI) convention.

**Figure 3:**
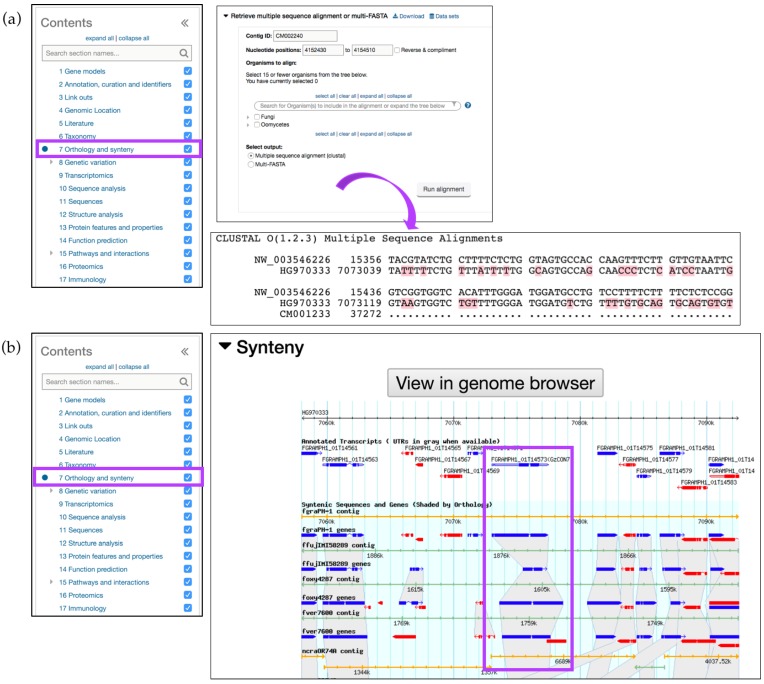
Simple searches and gene record pages, continued. *Orthology and Synteny* section (highlighted in purple) for the *F. graminearum* gene FGRAMPH1_01G14573. (http://fungidb.org/gene/FGRAMPH1_01G14573) (**a**) A Multiple Sequence Alignment (MSA) run within the gene record page against *Magnaporthe* and *Sordaria* sequences. Shown as CLUSTAL Omega output; (**b**) Syntenic orthologs can be previewed from the GBrowse window within the gene page. A separate session in GBrowse page can be deployed by clicking on the *View in genome browser* button. Syntenic regions for the gene are highlighted in purple.

The *Orthology and synteny* section provides a table of Orthologs and Paralogs within FungiDB (Figure 3a). This table is searchable and each ortholog in the table is hyperlinked to its gene page in FungiDB. An additional feature to interrogate sequence similarities between species of the same or different genera is offered via the *Retrieve multiple sequence alignment or multi-FASTA* tool. A user can select up to 15 organisms to perform multiple sequence alignment for a particular gene. This query uses pairwise Mercator [19] analysis combined with a Clustal Omega output format. A sequence alignment of FGRAMPH1_01G14573 with *Magnaporthe* and *Sordaria* species, selected from the *Organisms to align* parameter option, generated an output of genomic sequence IDs as identifiers. NW_003546226 and HG970333 identifiers belong to *F. graminearum* PH-1 and *Sordaria macrospora* genomes, respectively (Figure 3a). The gene record page for FGRAMPH1_01G14573 also contains a GBrowse display of synteny between related organisms with syntenic orthologs shaded in grey. An interactive display is also accessible via the *View in genome browser* button and highlighted in Figure 3b. 

The *Genetic Variation* section summarizes integrated FungiDB SNP data for a given region and classifies SNPs into groups based on the resulting effect on gene function: noncoding, nonsynonymous or synonymous, and nonsense nucleotide changes.

Genetic variation tracks can be explored in GBrowse and SNPs visualized by clicking on the *View in genome browser* button and activating appropriate tracks from the *Select Tracks* tab in GBrowse.

**Figure 4:**
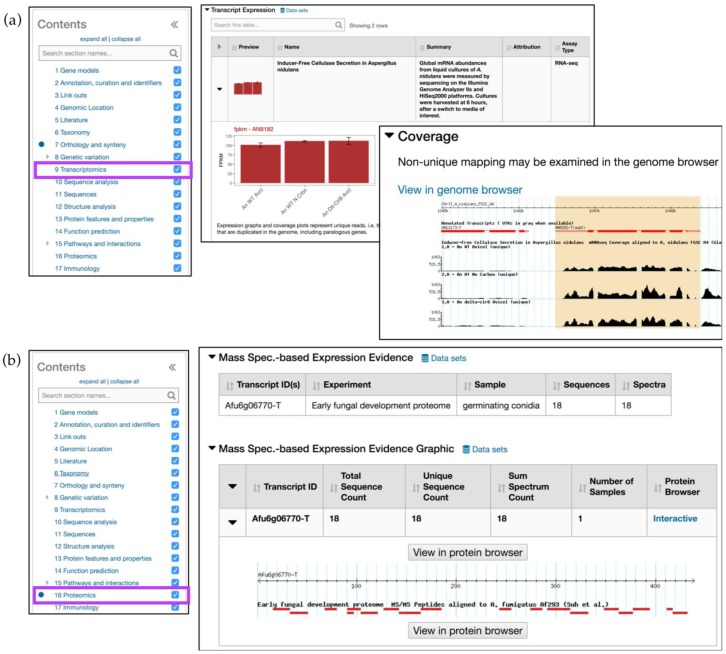
Simple searches and gene record pages, continued. (**a**) *Transcriptomics* menu (highlighted in purple) contains *Transcript Expression* records that show fpkm graphs for *A. nidulans* gene AN8182 (gene record page: http://fungidb.org/fungidb/app/record/gene/AN8182). Coverage plots (an inset to the *Transcript Expression* section) displays unique read alignments and a *View in genome browser* link out; (**b**) *Proteomics* menu (highlighted in purple) displays Mass Spec. Evidence for *A. fumigatus* gene Afu6g06770 (gene record page: http://fungidb.org/fungidb/app/record/gene/Afu6g06770). Proteomics tracks can be also visualized by clicking on the *View in protein browser* button.

The *Transcriptomics* section (RNA-Seq and microarray data) provides expandable rows with tabular data, summaries, coverage, expression graphs, and more. For example, the *Aspergillus nidulans* AN8182 gene record page demonstrates expression profiles for AN8182 where mRNA samples were collected from wild-type and *clr-2/clrB* mutants after a culture shift from sucrose/glucose to Avicel (crystalline cellulose) or no carbon media [20] (Figure 4a). The *Expression graph* (fpkm—AN8182) provides an overview of peaks detected in three treatments and *Coverage Section* shows uniquely mapped reads. When reads map to several genome locations and therefore could have been derived from multiple transcripts they are labeled as nonunique. Both unique and nonunique reads are now displayed in fpkm graphs and are available in GBrowse.

Next, the *Sequences*, *Sequence analysis, Structure*, and *Protein features and properties* menus offer DNA, RNA, and protein sequence information followed by records about known protein domains (InterPro), signal and Transmembrane predictions, BLASTP hits, BLAT hits against the nonredundant protein sequence database (GenBank), and other information. 

The *Function Prediction* section houses Gene Ontology assignments (downloaded from Gene Ontology databases and manually curated by FungiDB for a selected group of organisms). FungiDB gene pages also display GO Slim terms from the generic subset for each GO term associated with that gene. The GO assignments are followed by other functional data and datasets, including the *Pathways and Interactions* (*Metabolic Pathways* and *Metabolic Pathway Reactions* sections), *Proteomics evidence* (Mass Spectrometry-based expression and post-translational modification data in table and graph formats).

*Pathways and interactions* provide descriptions of metabolic pathways that are loaded into FungiDB from the KEGG [21] and MetaCyc [22] repositories. Genes are linked to individual pathways by EC numbers and clicking on any of the metabolic pathway links will navigate to an interactive metabolic pathway viewer where the user can explore individual reactions or export all known data for a given pathway.

The *Proteomics* section contains Mass Spec. evidence data and phosphoproteomics datasets. In Figure 4b, the gene records page for the *Aspergillus fumigatus (A. fumigatus)* gene Afu6g06770 demonstrates that the protein is found in germinating conidia. A summary table with sequence counts and a link out to peptide alignments against the reference genome can be viewed in GBrowse.

## 4. Creating in Silico Experiments via the FungiDB Query Interface

### 4.1. Mining Proteomic Datasets

FungiDB integrates proteomics data that are presented in several ways: peptides are mapped to genes, gene expression levels are loaded based on quantitative Mass Spec. data, and post-translationally modified amino acids are visualized as GBrowse tracks. 

Genes may be identified based on proteomics evidence by selecting one of the searches under the *Proteomics* category in the *Search for Genes* section on the home page. In the example described here, quantitative proteomics data from Suh *et al.* [23] are interrogated to identify *A. fumigatus* genes up-regulated in the growing conidia and then cross referenced with *Gene Models* data for *Exon Count* to identify genes with no more than 5 exons.

**Figure 5:**
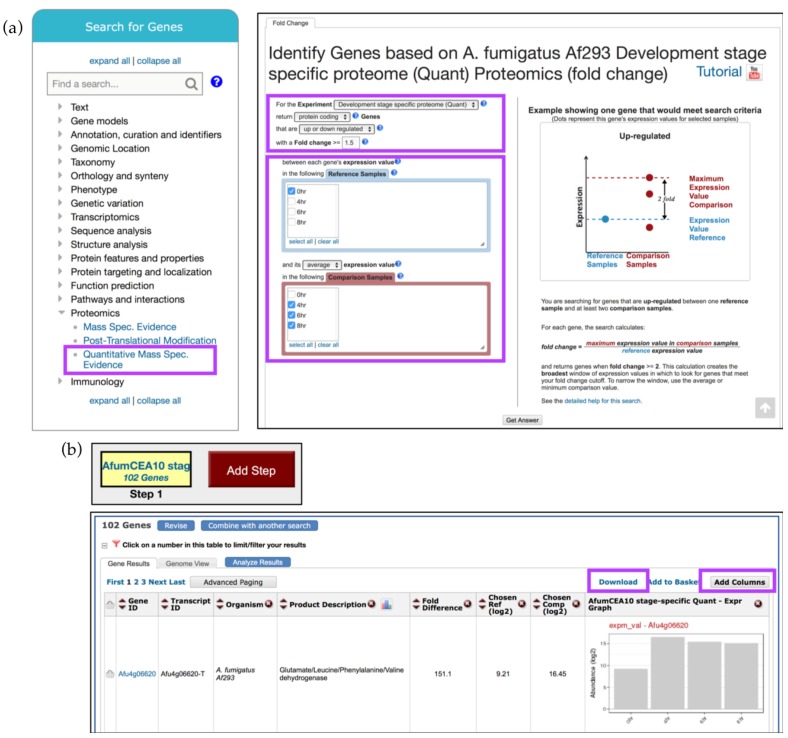
Quantitative Mass Spec. search examining up-regulated genes in *A. fumigatus* Af293 during conidial growth. (**a**) Select the *Quantitative Mass Spec. Evidence* menu (highlighted in purple) to access Suh *et al.* dataset and select *Reference* and *Comparison Samples* as shown to identify genes that are up-regulated throughout conidial development. The selection parameter areas are highlighted in purple; (**b**) Overview of the search strategy. Explore results, *Add Columns*, or *Download* results (highlighted in purple). Strategy in FungiDB: http://fungidb.org/fungidb/im.do?s=96851d3ef002899a.

The *Quantitative Mass Spec. Evidence* search can be accessed from the *Proteomics* section under the *Search for Genes* category (Figure 5a). Then, in the proteomics dataset selection window, click on the *Fold Change* (FC) button next to the Suh *et al.* study. To identify genes that are up-regulated by at least twofold in the 4–8 h time frame compared to the 0 h time point, set the regulation direction to *up-regulated*, modify the Fold Change parameter to 2, and select 0 h from the *Reference Samples* and 4, 6, and 8 h from the *Comparison Samples*, and click *Get Answer* (Figure 5a). 

The search strategy returned 102 genes that are up-regulated during conidial growth (Figure 5b). Examine or revise results, explore additional data by adding more columns via the *Add Columns* button, or export records via the *Download* link (Figure 5b and highlighted). 

**Figure 6:**
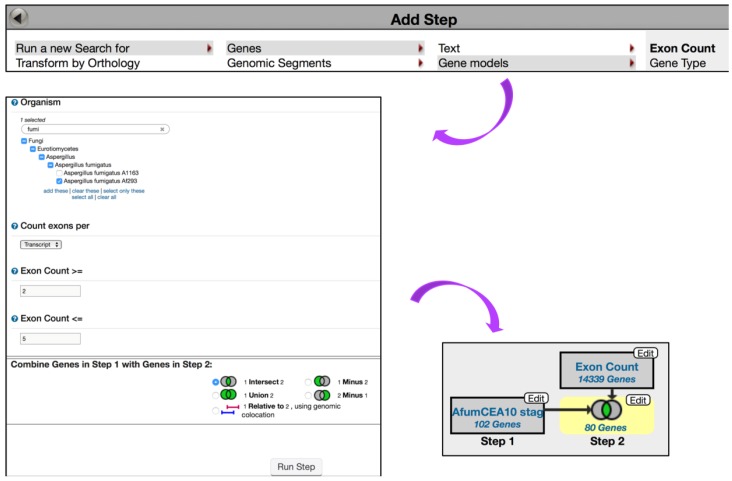
Quantitative Mass Spec. query examining up-regulated genes in *A. fumigatus* Af293 during conidial growth with at least 2 and no more than 5 exons. *Exon Count* search is deployed from the *Search for Genes* category, *Gene models* menu, *Exon Count* subcategory. Strategy in FungiDB: http://fungidb.org/fungidb/im.do?s=96851d3ef002899a.

To further expand this search strategy and determine how many of these genes have multiple exons, click on the *Add Step* button and choose to *Run a New Search for, Genes, Gene models, Exon Count* (min >= 2, max <= 5) (Figure 6)*.* Intersecting the second search with the first one returned 80 genes in *A. fumigatus*. 

### 4.2. Creating In Silico RNA-Seq Experiments

There are several ways to identify genes based on RNA-Seq Evidence. Depending on the type of integrated dataset, users can choose to analyze a dataset using Differential Expression (DE), Fold Change (FC), or Percentile (P). In this example, we will query a dataset published by Chen *et al.* [24] and compare transcriptomes of two strains of *Cryptococcus neoformans*, a fungal organism causing fungal meningitis and high mortality rates in immunocompromised patients [25,26]. The HC1 and G0 strains were isolated from the cerebrospinal fluid (*in vivo* CSF) of patients in the U.S. and Uganda, respectively, and then also incubated in pooled human CSF (*ex vivo* CSF) and in yeast extract–peptone–dextrose (YPD). 

**Figure 7:**
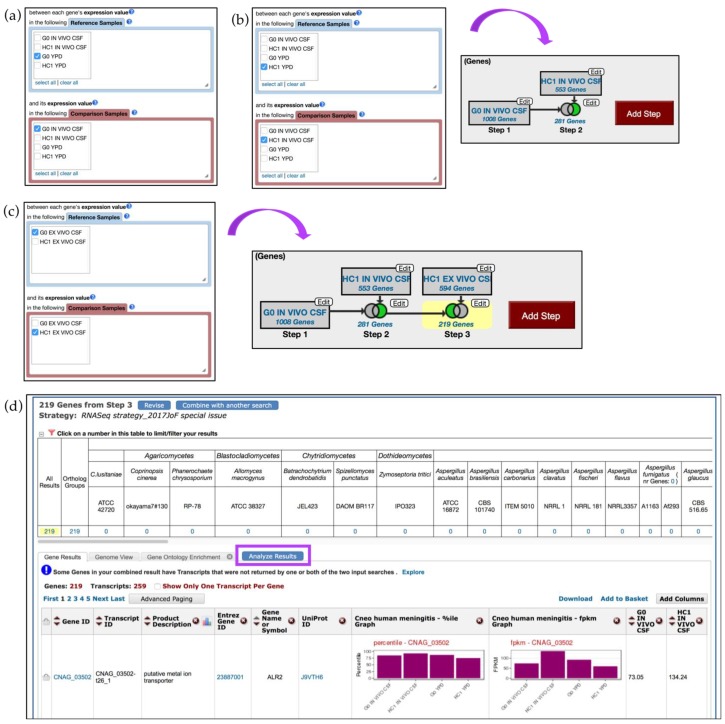
RNA-Seq query using *C. neoformans* single-read and paired-end datasets by Chen *et al.* [24] (**a**) RNA-Seq query on single-read data is initiated from the *Search for Genes* panel, *Transcriptomics* menu. Step 1 selects for two-fold up-regulated genes in *G0* in vivo *CSF* compared with in the *G0 YPD* reference sample; (**b**) Step 2 selects for *HC1* genes expressed in vivo *CSF* (using HC1 *YPD* sample as reference). Choose the *2 minus 1* intersect parameter to set up conditions for the final search; (**c**) Add Step and repeat steps as before but this time select the paired-end dataset. Step 3 identifies HC1 genes up-regulated in vivo *CSF* compared to ex vivo *CSF*. Choose the *1 minus 2* intersect parameter for the third step; (**d**) Results table for Step 3. Clicking on the *Analyze Results* tab (highlighted in purple) will redirect users to enrichment analysis using *Gene Ontology*, *Metabolic Pathway*, and *Word Enrichment* tools. Strategy in FungiDB http://fungidb.org/fungidb/im.do?s=892406814924ecdd.

The strategy described below compares transcriptomes of two strains and aims to identify genes that are up-regulated in the HC1 strain compared with in the G0 strain (in vivo CSF) (Step 1–2) and then identifies those genes that are specific for in vivo expression in the HC1 strain (Step 3) (Figure 7). 

To initiate an *in silico* experiment, click on the *Transcriptomics* category on the home page or in the drop-down menu from the grey main menu and then navigate to the *RNA Seq Evidence* page. From the list of available studies, click on the fold change (FC) button in blue for the *Cryptococcus neoformans transcriptome at the site of human meningitis (single-read data)* dataset. Next, select to identify genes that are up-regulated by twofold between each gene’s expression value in the *G0 YPD* sample (*Reference Sample*) and its expression in the *G0* in vivo *CSF* sample (*Comparison Sample*) (Figure 7a). 

Next, click on the *Add Step* button, *Run a new Search for, Genes*, *Transcriptomics*, *RNA Seq Evidence* and repeat previous steps but this time identify genes that are twofold up-regulated in *HC1* in vivo *CSF* (*Comparison sample*) versus in *HC1 YPD* (*Reference sample*). To set conditions for the next search, select the *2 minus 1* intersect option from the four available set operations (Figure 7b). As a result, Step 2 returned 281 genes that are up-regulated in *HC1* in vivo *CSF* but not in *G0* in vivo *CSF*. 

To further delineate in-vivo-specific genes in HC1 strain, one can create an additional step to eliminate genes that were expressed in ex vivo conditions in the HC1 strain. Click on the *Add Step* button, navigate to the RNA-Seq datasets selection window and this time choose the paired-end dataset from Chen *et al.* instead of the single-read data. Configure the search to return genes that are twofold up-regulated in the *HC1* ex vivo *CSF* (*Comparison sample*) versus in the *G0* ex vivo *CSF* sample (*Reference sample*). Choose the *2 minus 3* option and click on the *Run Step* button (Figure 7c). 

The strategy described above used two different types of data (single-read and paired-end RNA-Seq data) to examine differential gene expression. To further investigate results of gene-specific queries, users can take advantage of a number of enrichment tools located under the *Analyze Results* tab in blue (Figure 7d), which is reviewed further in the text.

## 5. Additional Tools for Enrichment and Data Analysis

When working with a list of genes such as RNA-Seq results (as described in Figure 6) or user-uploaded gene lists, one can perform several enrichment analyses to further characterize results into functional categories. Enrichment analysis can be accessed via the blue *Analyze Results* tab and it includes *Gene Ontology* [27,28], *Metabolic Pathway* [21,22], and *Word Enrichment* tools. The three types of analysis apply Fisher’s Exact test to evaluate ontology terms, over-represented pathways, and product description terms. Enrichment is carried out using a Fisher’s Exact test with the background defined as all genes from the organism being queried. The *p*-values corrected for multiple testing are provided using both the Benjamini–Hochberg false discovery rate method [29] and the Bonferroni method [30].

### 5.1. GO Term Enrichment and Visualization

**Figure 8:**
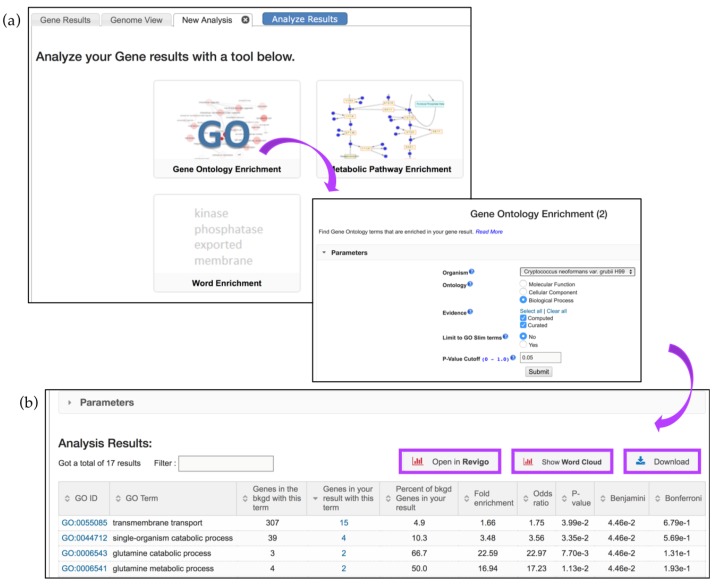
Gene enrichment analysis tools. (**a**) Clicking on the *GO*—*Gene Ontology Enrichment* button deploys GO enrichment that can be limited based on the following parameters: ontology aspects, GO evidence (*Computed* vs. *Curated*), and *p*-value (0.05 default), or GO Slim terms; (**b**) The enrichment results can be further visualized via Reduce + Visualize Gene Ontology (REViGO), Word Cloud image, or downloaded (highlighted in purple).

To determine if a gene list contains enriched functions, a GO enrichment analysis may be performed. Continuing with the RNA-Seq example described in Figure 7, click on the *Analyze Results* tab, and then select the *Gene Ontology Enrichment* button (Figure 8a). 

GO enrichment parameters allow users to limit their analysis on either *Curated* or *Computed* annotations, or both. Those with a GO evidence code inferred from electronic annotation (IEA) are denoted *Computed*, while all others have some degree of curation. Users can also choose to show results for the following functional aspects of the GO ontology: molecular function, cellular component, and biological processes, as well as set a custom *p*-value cut-off. When the GO slim option is chosen, both the genes of interest and the background are limited to GO terms that are part of the generic GO Slim subset (Figure 8a). Users may download a GO enrichment table (with the Gene IDs for each GO term added) as well as view and download a word cloud produced via the GO Summaries R package [31] (Figure 8b, highlighted in purple).

The Analysis Results table from the GO enrichment focusing on biological function shows enrichment for transmembrane transport genes. The table contains columns with GO IDs and GO terms along with the number of genes in the background and those specific to the RNA-Seq analysis results presented. Several additional statistical measurements are also included and are defined below:

- *Fold enrichment*—The ratio of the proportion of genes in the list of interest with a specific GO term over the proportion of genes in the background with that term;
- *Odds ratio*—Determines if the odds of the GO term appearing in the list of interest are the same as that for the background list;
- *p-value*—Assumptions under a null hypothesis, the probability of getting a result that is equal or greater than what was observed;
- *Benjamini–Hochburg* false discovery rate—A method for controlling false discovery rates for type 1 errors;
- *Bonferroni-adjusted p-values*—A method for correcting significance based on multiple comparisons;

GO enrichment analysis returned genes enriched in transmembrane transport (GO:0055085), single-organism catabolic process (GO:0044712), etc. (Figure 8b). These and other terms can be taken to an external REViGO website [32] by clicking on the *Open in Revigo* button. REViGO visualizes the GO enrichment results while removing redundant GO terms. The resulting similarity-based scatterplots are color-coded and provide interactive graphs and tag clouds as alternative visualizations. More information is available at the Revigo website: http://revigo.irb.hr.

### 5.2. Metabolic Pathway Enrichment

The Metabolic Pathway Enrichment tool allows the user to determine whether particular metabolic pathways are over-represented in a subset of genes from an organism compared to the background. Described here is a search query using a list of genes from a publication studying pathogen adaptation responses in *Paracoccidioides brasiliensis* (*P. brasiliensis*), one of the most frequent causative agents of systemic mycoses in Latin America [33]. 

When fungal conidia of *P. brasiliensis* gain access to host tissues and organs they differentiate into a parasitic form that undergoes morphological changes conferring resistance to immune responses of the host. Alternative carbon metabolism is particularly important for the survival and virulence of this fungal pathogen within the host, Lima and colleagues reported that thirty-one genes of *P. brasiliensis* were up-regulated under carbon starvation conditions as determined by both RNA-Seq and proteomics studies [33].

**Figure 9:**
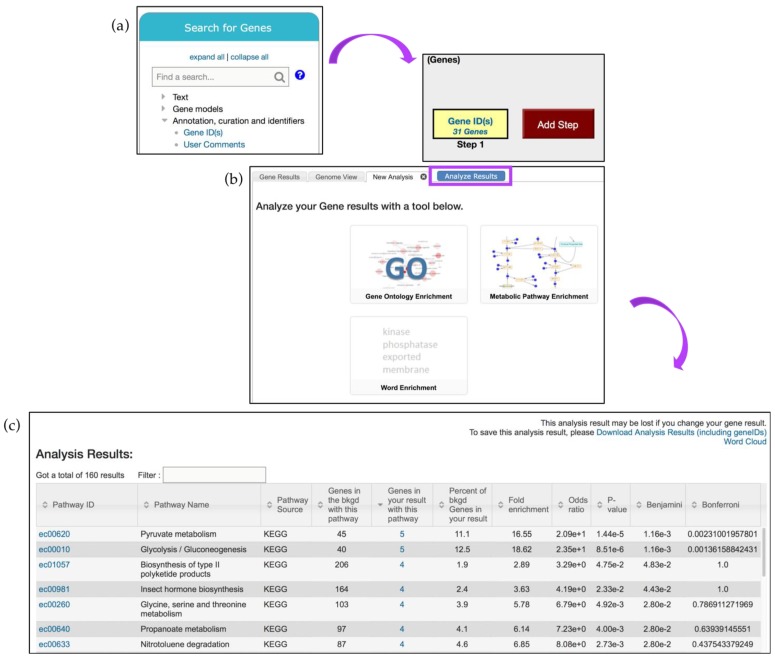
Metabolic pathway enrichment analysis. (**a**) A list of 31 genes is analyzed via the *Gene IDs* tool (from the *Search for Genes* panel) and the (**b**) the *Metabolic Pathway Enrichment* tool from the *Analyze Results* tab (highlighted in purple) (**c**) Metabolic Pathway Enrichment analysis results page. Individual *Pathway ID* (e.g., ec00620) is linked to the interactive CytoScape interface. Strategy in FungiDB: http://fungidb.org/fungidb/im.do?s=6825e606be957ead.

Here, thirty-one genes described above are identified using the *Gene ID(s)* search, deployed via the *Search for Genes* menu and the *Annotation, curation and identifiers* submenu (Figure 9a). Next, deploy the *Metabolic Pathway Enrichment* tool via the *Analyze Results* tab, use *KEGG* pathway enrichment only, and then click *Submit* to start the analysis (Figure 9b). The analysis may take some time to run depending on the size of the gene list. Results will be presented in a new tab (Figure 9c).

**Figure 10:**
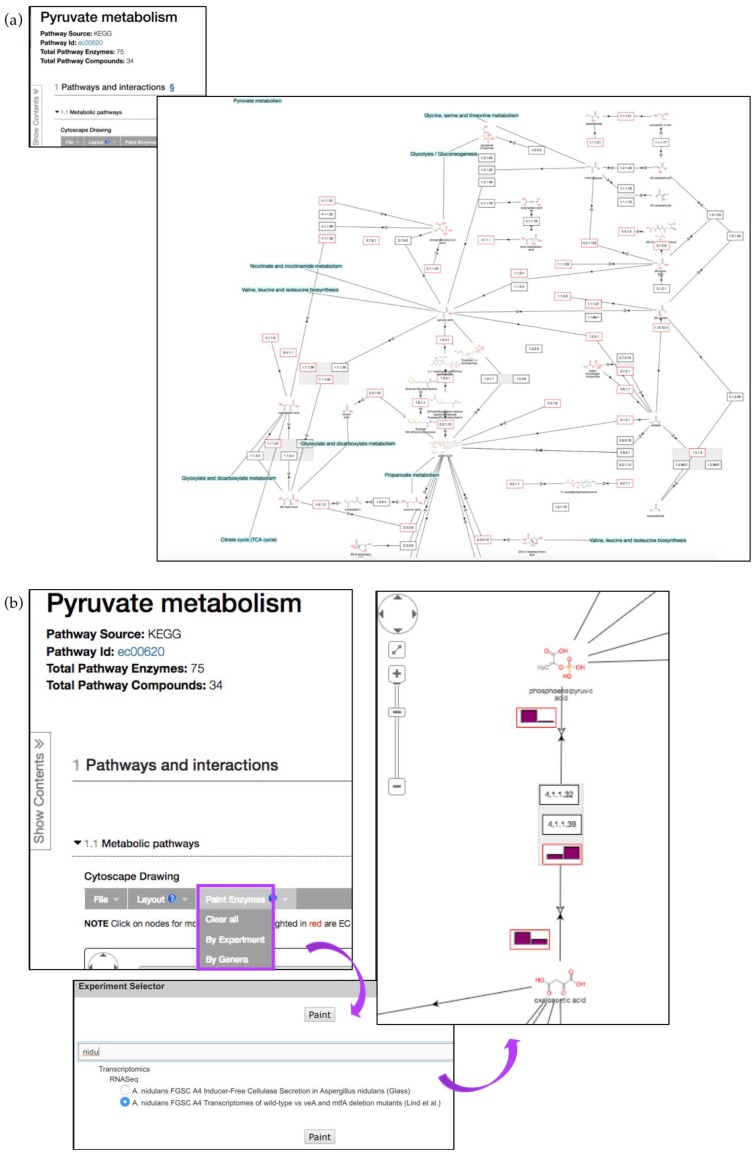
Interactive pathway map for KEGG ec00620 (Pyruvate metabolism). (**a**) Enzyme nodes that are represented by a gene in the database are outlined in red. Clicking on enzyme or compound nodes reveals more information about corresponding entities (**b**) Pyruvate metabolism pathway with RNA-Seq fpkm values painted above individual pathway nodes (*A. nidulans* transcriptomics data) via the *Paint Enzyme* menu (highlighted in purple).

KEGG pathway enrichment on the gene list described above highlighted enrichment in carbohydrate, amino acid, and lipid metabolism, corroborating the findings described in the manuscript [33]. These pathways can be explored further by clicking on the link in the *Pathway ID* column. The pyruvate metabolism pathway (ec00620) is one of the pathways enriched amongst *P. brasiliensis* genes that were up-regulated during starvation (Figure 10a). 

Cytoscape interactive map offers zoom-in, zoom-out, and reposition capabilities as well as additional pathway node information. Enzyme nodes can be painted with corresponding available transcriptomic or proteomic data using the *Paint Enzymes* menu. For instance, the pyruvate metabolism pathway is also present in *Aspergillus*. The pathway record pages can be accessed from the gene record page for *A. nidulans* pycA gene AN4462. Gene expression profiles can then be painted onto individual nodes in *A. nidulans* via the *Paint Enzyme* menu option (Figure 10b). Any gene that does not have expression data for the chosen organism/experiment will have the text “None” in the enzyme box. 

## 6. Data Mining via the *Search for Other Data Types* Panel 

### 6.1. Identifying SNPs between Fungal Isolates Collected in Various Geographical Areas

The example described below identifies SNPs in *Coccidioides posadasii (C. posadasii)* str. Silveira isolates collected from patients with Coccidioidomycosis in the U.S. and Latin America [34]. Coccidioidomycosis, also known as Valley fever, is a fungal disease caused by two closely related species—*C. immitis* and *C. posadasii* [34,35]. The disease is associated with high morbidity and mortality rates and affects tens of thousands of people each year. The two fungal species are endemic to several regions in the Western Hemisphere, but recent epidemiological and population studies suggest that the geographic range of these fungal species is becoming wider [36]. 

**Figure 11:**
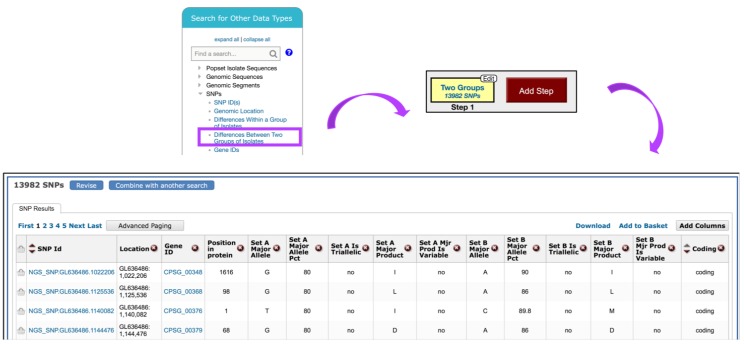
Search for SNPs via the *Differences Between Two Groups of Isolates* option. SNP search is initiated from the *Search for Other Data Types* panel on the home page (highlighted in purple). The SNPs results table shown is sorted by *Coding* column.

To identify SNPs between isolates collected in *Guatemala* and the *U.S.*, navigate to the *Identify SNPs based on Differences Between Two Groups of Isolates* from the *Search for Other Data Types panel* (Figure 11). In the resulting window, scroll through the metadata options on the left and make appropriate *Geographic Location* selections from the *Host* section of *Characteristic* separately for Set A and Set B isolates. Set A isolates should be set to *Guatemala* and Set B to the *United States of America*. All other parameters for both sets should be left as default. 

The search strategy returns SNPs rather than genes, which are classified by genomic location within the results table. When individual SNPs fall within a gene, the corresponding Gene ID is listed next to the SNP record (Figure 11). 

**Figure 12:**
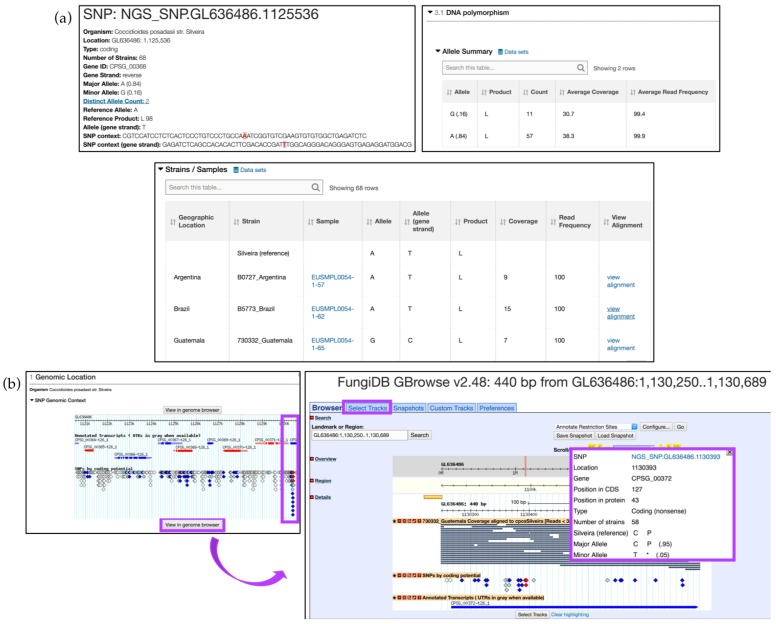
Search for SNPs via the *Differences Between Two Groups of Isolates* option, continued. (**a**) SNP record for SNP.GL636486.1125536 corresponding to the CPSG_00368 gene. *DNA polymorphism* and SNP categorization based on *Strains/Samples* are shown; (**b**) Explore SNPs in FungiDB GBrowse. SNPs are color coded based on their effect on gene function (e.g., nonsense SNPs are shown as red diamonds-highlighted in purple). Click on the *View in genome browser* button to be re-directed to GBrowse. Individual reads can be activated from the *Select Tracks* tab (highlighted in purple). Hover over a SNP to activate a pop-up box with additional information (highlighted in purple). Strategy in FungiDB: http://fungidb.org/fungidb/im.do?s=564aa900d161cbbb.

To examine an SNP record page, sort the results by the *Coding* polymorphisms and click on the SNP.GL636486.1125536 SNP in the CPSG_00368 gene, located within the third page of the results. SNP location, allele summary, associated Gene ID, and major and minor allele records can be found at the top of the page, followed by DNA polymorphism summary and an SNP records table that is searchable by isolates (Figure 12a). Genomic location, SNP type, and aligned reads can be displayed in GBrowse by clicking on the *View in genome browser* button (Figure 12b). The SNP track can be activated from the *Select Tracks* tab by selecting *SNPs by coding potential* under *DNA polymorphism* in the *Genetic variation* section. Hover over SNPs labeled with red diamonds (nonsense SNPs) to get more information. Note that individual reads comprising SNP data can be activated from the *Genetic variation* section by selecting either all or specific isolates listed under the *Aligned Genomic Sequence Reads for C. posadasii* str. Silveira.

### 6.2. Utilizing the Genomic Location Search and Genomic Colocation Tool in Custom Queries

The *Genomic Location* search tool empowers researchers to create custom queries for genomic regions of interest (e.g., custom sets of coordinates). The strategy described below investigates the effect of LTR/Gypsy Transposable Elements (TEs) (loci provided by Theo Kirkland; unpublished data) on expression of neighboring genes in *C. immitis* RS. This search strategy takes advantage of two types of queries reviewed above: *Search Other Data Types* and *Search for Genes*. 

**Figure 13:**
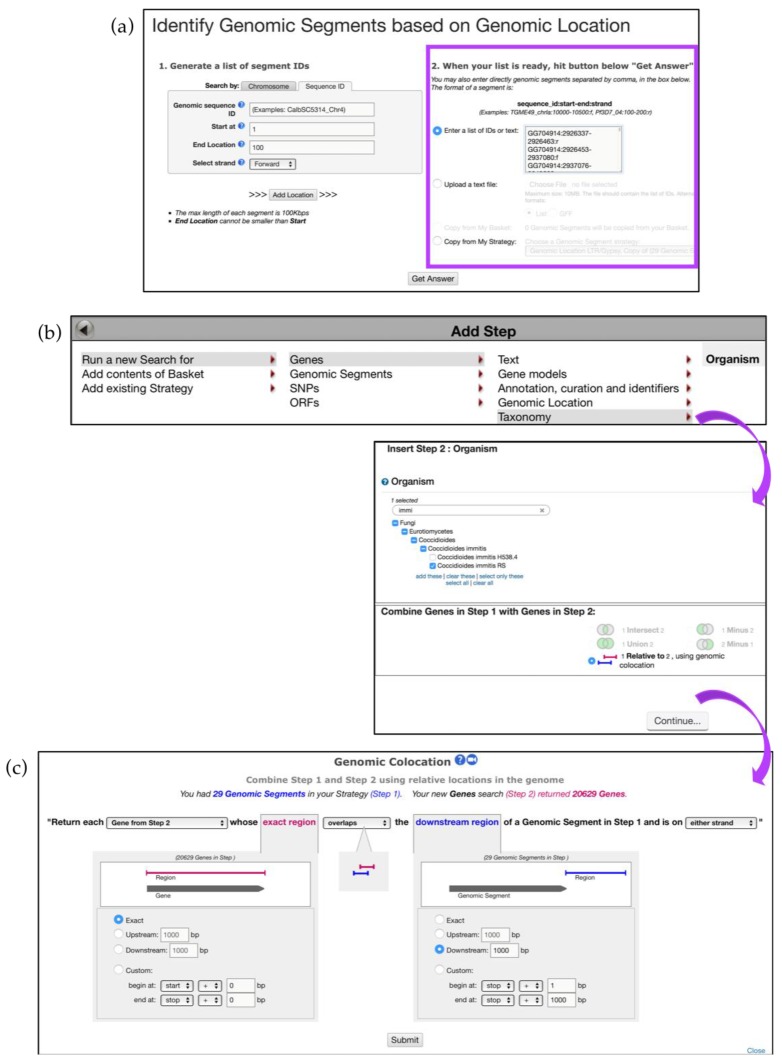
Using *Genomic Location* search to discover impact of Transposable Elements (TEs) on gene expression. (**a**) Identify TEs based on coordinates formatted to the accepted convention (e.g., *sequence_id:start-end:strand). Paste a list of genomic coordinates in the highlighted section*—Step 1 (**b**) *Add Step* and *Run a new Search* using a *Taxonomy* tool and identify *C. immitis genes that* are (**c**) 1000bp downstream of the previously identified TE elements—Step 2. Strategy in FungiDB: http://fungidb.org/fungidb/im.do?s=67dcb3bb26a92111.

Twenty-nine LTR/Gypsy genomic coordinates were entered in the search window initiated via the *Search for Other Data Types, Genomic Segments*, *Genomic Location* selection menus (Figure 13a). All genomic locations were previously formatted to conform to the following format: *sequence_id:start-end:strand* (e.g., *GG704914:2926337-2926463:r*). 

Initiate a new search and *Run a new Search for Genes, Taxonomy, Organism,* and choose *C. immitis* and the *1 relative to 2* search option (Figure 13b). The next screen will bring up the *Genomic Colocation* tool that requires selection of several search parameters. Choose to *Return each Gene from Step 2 whose exact region overlaps with downstream region of a Genomic Segments in Step 1 and is on either strand.* Look for genes that are located 1000 bp downstream of the segments identified in Step 1 (Figure 13c).

**Figure 14:**
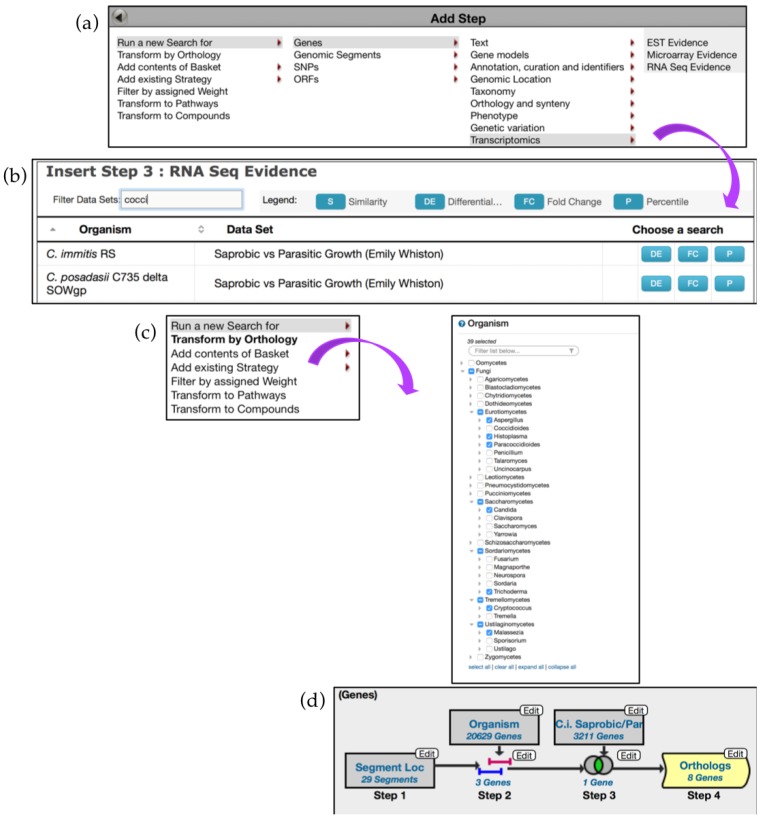
Using *Genomic Location* search to discover impact of Transposable Elements (TEs) on gene expression, continued. (**a**,**b**) *Add Step* to mine transcriptomics data for *C. immitis*—Step 3 (**c**) Step 4: *Add step* to deploy the *Transform by Orthology* tool. (**d**) Overview of the search strategy. The shape of the search box highlighted in yellow is different to reflect changes in fungal species/genera. Strategy in FungiDB: http://fungidb.org/fungidb/im.do?s=67dcb3bb26a92111.

The second search returned three genes. Next, examine transcriptomic records integrated in FungiDB by adding a dataset from the *RNA Seq Evidence menu* for *C. immitis (Saprobic vs Parasitic Growth (Emily Whiston)) dataset* (Figure 14a,b)*.* Based on the search criteria entered, only one gene was identified—CIMG_13457, a hypothetical protein. One can try to identify orthologs of this gene in other species by adding another step and deploying the *Transform by Orthology* tool for *Eurotiomycetes*, *Saccharomycetes (Candida)*, *Sordariomycetes (Trichoderma)*, *Tremellomycetes (Cryptococcus)*, and *Ustilaginomycetes (Malassezia)* (Figure 14c). The search identified eight orthologs in other pathogens with the majority occurring in *Histoplasma* species and a single ortholog in *P. brasiliensis* Pb03 (Figure 14d). 

### 6.3. Searching for Fungal-Specific Motifs

Proteins which perform similar functions often contain conserved sequences such as protein domains or motifs that facilitate important functions such as enzyme binding, post-translational modifications, or cellular trafficking. FungiDB offers tools for finding peptide and DNA motifs in target sequences. The strategy described below takes advantage of the *Protein Motif Pattern* search tool to identify effector proteins in the wheat stem rust fungi *Puccinia gramminis* f. sp. tritici. 

Rust fungi are pathogens of crops that depend on developing haustoria in the living plant cells to support nutrient absorption and sporulation. A new class of Y/F/WxC effector proteins was reported in haustoria-producing plant pathogens by Godfrey *et al.* [37]. Before starting a search, the amino acid pattern of interest must be converted into a Perl-style regular expression. Using Perl characters (Table 1), the Y/F/WxC motif is expressed as {0,42}[YFW][^C]C, where [YFW] is a 3-amino acid peptide that has an aromatic amino acid, followed by any amino acid except a cysteine, followed by a cysteine, with any amino acid occurring between 0 and 42 times before the 3-amino acid motif.

**Figure 15:**
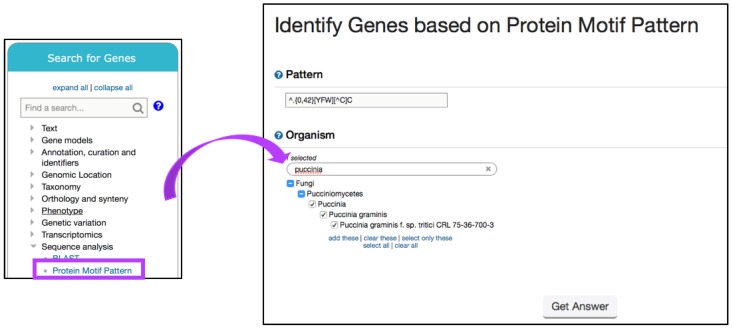
Identifying genes via the *Motif Pattern* tool. Shown is a search for the effector protein motif in *P. gramminis* using a regular expression (Step 1). Strategy in FungiDB: http://fungidb.org/fungidb/im.do?s=4e9454cd679286de.

**Figure 16:**
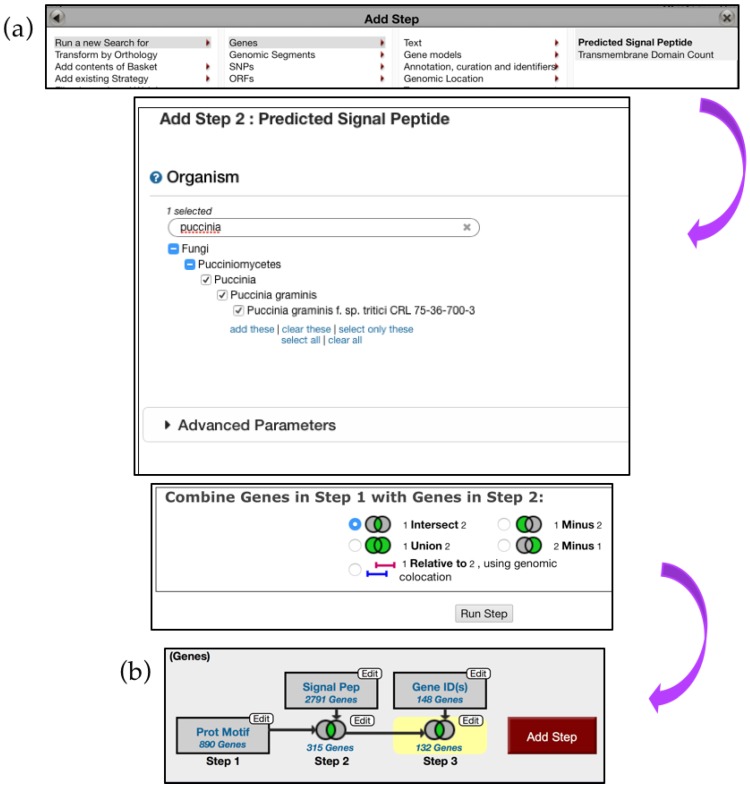
Identifying genes via the *Motif Pattern* tool, continued. (**a**) *Predicted Signal Peptides* search deployed via the *Add Step* function (Step 2) (**b**) Overview of the search strategy. Search results from Step 2 are intersected with 148 genes reported by Godfrey *et al.* (Step 3) Strategy in FungiDB: http://fungidb.org/fungidb/im.do?s=4e9454cd679286de.

Next, navigate to the *Search for Genes* panel, *Sequence analysis* menu, and then click on the *Protein Motif Pattern* submenu (Figure 15). In the *Identify Genes Based on Protein Motif Pattern* page, enter {0,42}[YFW][^C]C in the *Pattern* parameter and select *P. gramminis* from the list of fungal organisms. *Run a new Search for Genes* based on *Protein Targeting and Localization* and identify if any of the genes have a *Predicted Signal Peptide* (Figure 16a). Choosing to intersect your search with previous results will identify several hundred proteins with predicted Y/F/WxC motif and signal peptide domains. One hundred and thirty-two of these genes matched the BLAST analysis reported by Godfrey *et al.* [37] (Figure 16b). Closer examination of additional gene hits would be needed to determine their biological relevance for plant–fungal interactions.

## 7. EuPathDB/FungiDB Galaxy Workspace

EuPathDB Galaxy-based [38] workspace offers preloaded genomes, private data analysis and display, and the ability to share and export analysis results [2]. Developed in partnership with Globus Genomics [39] and accessed through the *Analyze My Experiment* tab on the home page of FungiDB, the EuPathDB Galaxy instance houses a large variety of bioinformatics tools. Users can create their own private Galaxy workspace by creating a Globus account through FungiDB or any other EuPathDB database. After an account is created, sample workflows can be imported for editing, and new custom workflows can be created which can be stored privately or shared with the wider community or between EuPathDB Galaxy users. Results can be exported or shared with collaborators, but the instance is not meant as a repository for long-term data storage. All genomes available in FungiDB/EuPathDB sites are already preloaded into the EuPathDB Galaxy instance and, alongside these, users can upload their own data for analysis.

### Running a Preconfigured (Sample) Workflow

The EuPathDB Galaxy workspace has several workflows for RNA-Seq analysis and SNP calling (available from the EuPathDB Galaxy Welcome page). Each pipeline can be initiated directly from the welcome page; however, necessary input files must be uploaded prior to deploying a preconfigured workflow.

The analysis described here uses files obtained from the EBI BioProject PRJEB13569, which is a RNA-Seq dataset deposited by the Wheat Open Blast initiative [40]. This dataset comprises RNA-Seq samples collected from wheat leaf blades harvested from wheat-blast-infected fields in Bangladesh [41]. Wheat blast is a fungal disease caused by *Magnaporthe oryzae* (*M. oryzae*)*,* a plant pathogen threatening crops globally. 

**Figure 17:**
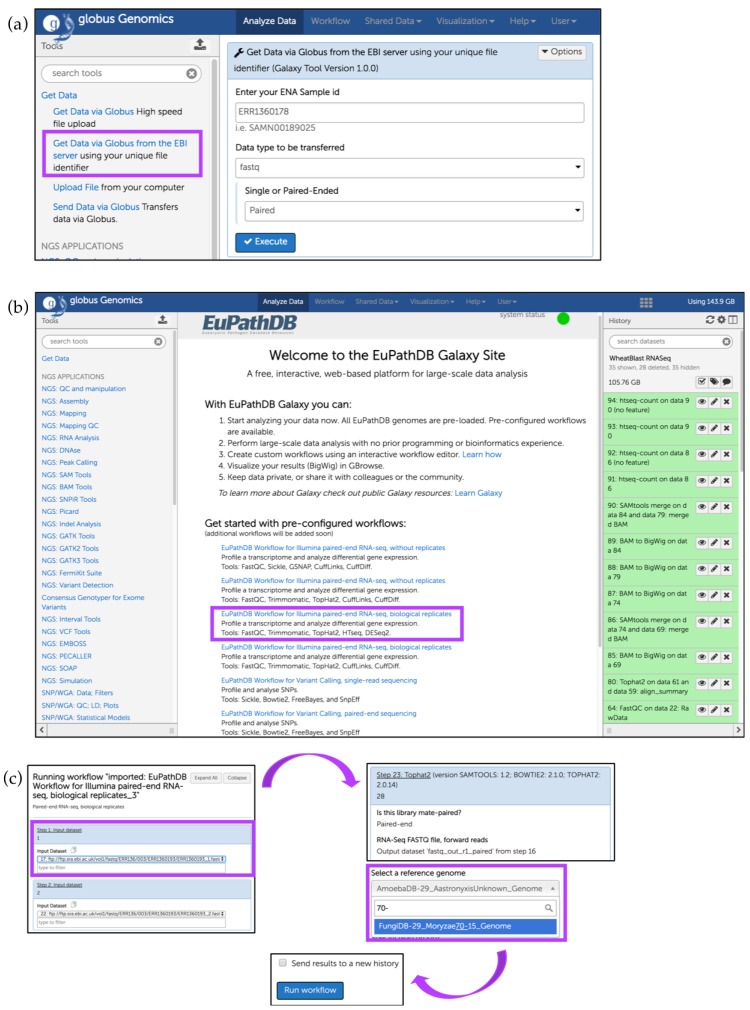
Running a preconfigured workflow for paired-end RNA-Seq data with biological replicates. (**a**) Upload files from EBI server (highlighted in purple); (**b**) Initiate a sample workflow by clicking on the corresponding workflow link (highlighted); (**c**) Choose preloaded paired datasets from the *Input dataset* drop down menu (highlighted). Some tools (e.g., Tophat2) require specification of the reference genome (highlighted) which will be used to map reads. Use search box to find and select the reference genome. Click on *Run workflow* to initiate the analysis.

To load the following files into the Galaxy instance, click on the *Get Data* link in the *Tools* section, select the *Get Data via Globus from the EBI server* option (Figure 17a), and enter Sample IDs. As an example, we will use two Asymptomatic samples F12 (Run accession numbers: ERR1360178, ERR1360179) and Symptomatic samples 12 (Run accession numbers: ERR1360192, ERR1360193). Specify *paired-end* sequencing for each accession number and click *Execute*. 

**Figure 18:**
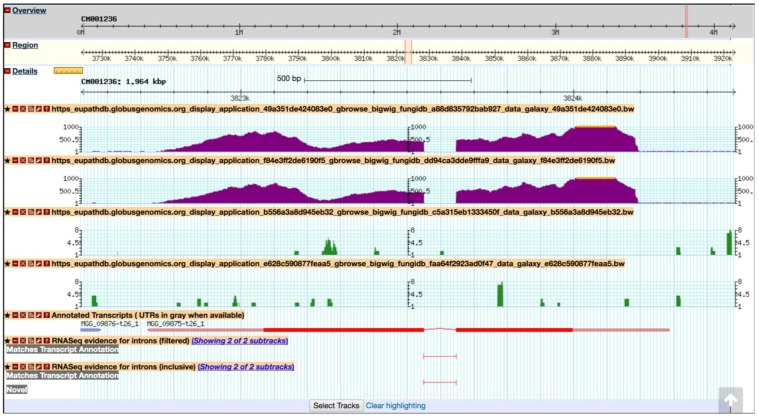
Importing BigWig files into the FungiDB GBrowse from the EuPathDB Galaxy instance. The data is scaled to local min/max. Shown are tracks from symptomatic and asymptomatic samples (both replicates), followed by Annotated Transcripts and FungiDB pre-configured RNASeq evidence tracks.

After all samples have been loaded successfully (highlighted in green), click on *Analyze Data* at the top of the EuPathDB Galaxy screen to return to the main page and click on the third workflow within the sample workflows list (Figure 17b); choose input datasets so that each pair forms a paired-end sample. This workflow uses FastQC [42], Trimmomatic [43], TopHat2 [44], HTseq-count [45], and DESeq2 [46]. Choose *M. oryzae* 70–15 genome for all workflow steps when prompted and then click on the *Run workflow* button (Figure 17c). To send this run into a new history, check the box *Send results to a new history* (Figure 17c). After the workflow has finished running, users can visualize the expression graphs (as BigWig files) in FungiDB GBrowse (Figure 18). To import BigWig files into FungiDB GBrowse, click on the *Display in FungiDB GBrowse* link, which will become visible once a user clicks to expand the *Bam to BigWig* step in the workflow EuPathDb Galaxy history section on the right (Figure 17b).

**Figure 19:**
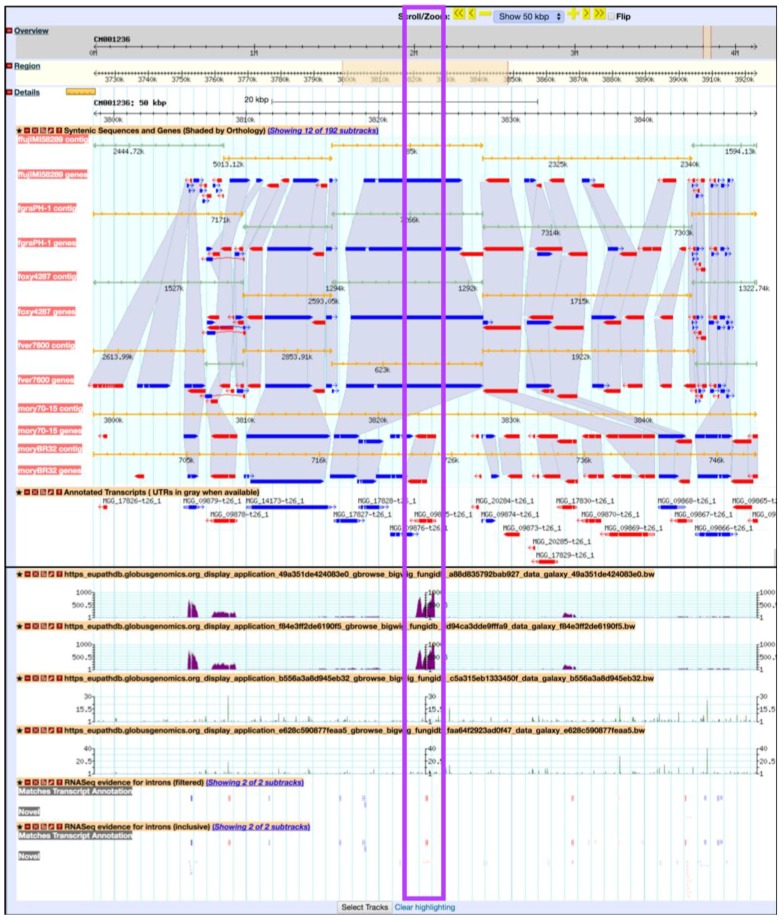
Viewing BigWig files in the FungiDB GBrowse. Shown are synteny tracks for selected Sordariomycetes (highlighted in purple). Tracks can be activated from the *Orthology and synteny* section, which is located under the *Select Tracks* tab.

Previously, Mathioni *et al.* [47] reported that *MAS3*, a *CAS1*-like domain-containing protein, was implicated in virulence in the blast fungus *M. oryzae* infecting barley. To determine if this gene is also up-regulated during infection in wheat in the RNA-Seq samples analyzed here, navigate to *MAS3* gene (MGG_09875) by entering the following coordinates in the Landmark or region search window in GBrowse: CM001236:3,822,721.3,824,284. As a result, the GBrowse window along with all corresponding BigWig tracks will be rescaled to the region specified. Next, zoom out to 50 kbp and deploy the synteny option for Sordariomycetes from the *Select Tracks* tab. As demonstrated in Figure 19, MGG_09875 is syntenic with an *M. oryzae* BR32 gene as one would expect; however, it is lacking syntenic orthologs in other species. To access nonsyntenic orthologs of *MAS3* in other species, navigate to the *Orthology and Synteny* section within the gene record page.

## 8. Retrieve Sequences via *Sequence Retrieval Tool*

**Figure 20:**
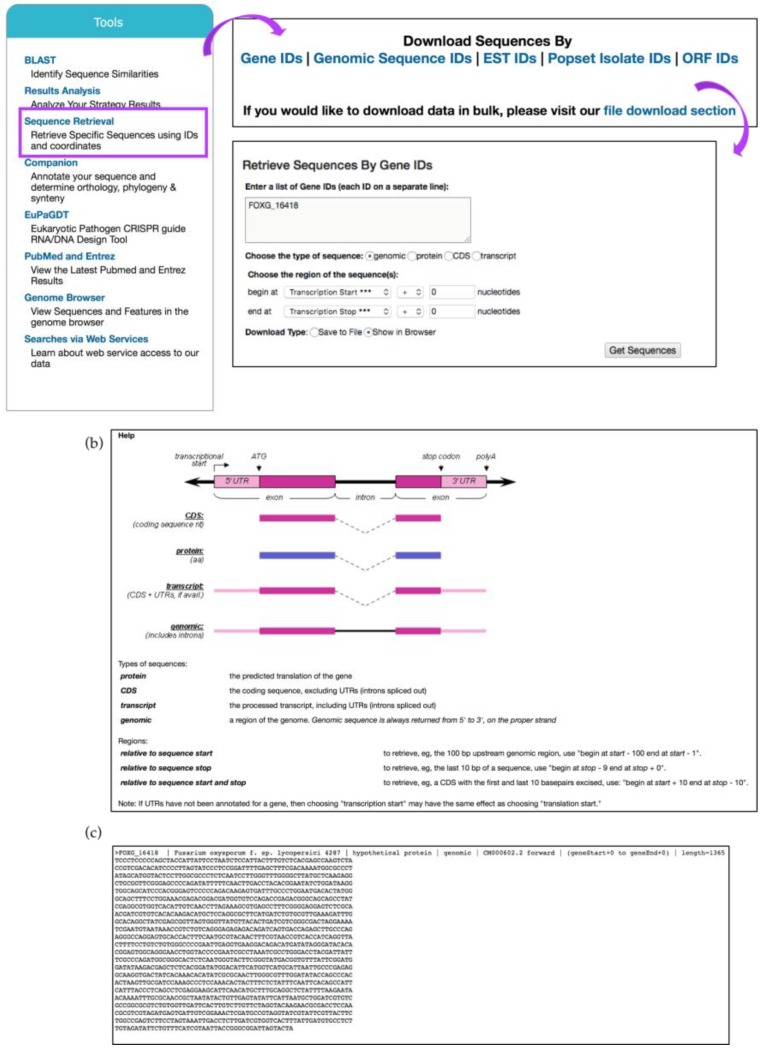
*Sequence Retrieval Tool*. (**a**) The tool can be initiated from the *Tools* component of the main page (highlighted in purple). Retrieving Sequences by Gene IDs is shown for gene FOXG_16418. Additional retrieval features are available: *Retrieve Sequences By Genomic Sequence IDs*, *Retrieve Sequences By EST IDs*, *Retrieve Sequences By Popset Isolate IDs*, *Retrieve Sequences By Open Reading Frame IDs*; (**b**) *Help* menu at the bottom of the page offers guidance in identification and selection of transcriptional and translation start and stop sites; (**c**) Browser view of the results showing FASTA sequence for the genomic sequence for gene FOXG_16418.

The *Sequence Retrieval Tool* (SRT) retrieves sequences in FASTA format using genome or proteome coordinates (Figure 20). SRT is accessible from the *Tools* component on the home page or from the *Tools* tab within the main grey main menu. This tool works independently from the data mining infrastructure used when creating queries and results can be shown in the browser or downloaded as a text file. The tool can be further customized by changing transcription/translation start and stop site settings (Figure 20a,b). *Fusarium oxysporum* f.sp. lycopersici 4287 is an important tomato pathogen. The FOXG_16418 gene encodes an effector protein that is required for virulence. To retrieve the genomic sequence of this gene, enter the gene ID in the text box within the *Retrieve Sequences By Gene IDs* window and select *genomic* sequence type. 

## 9. Empowering Users to Improve Genome Annotation

FungiDB has nearly 100 genomes and counting. Several *Aspergillus* species (*A. fumigatus* Af293 and *A. nidulans* FGSC A4), *Cryptococcus* (*C. neoformans* JEC21 and H99), and *Neurospora crassa* are under active curation (via Chado relational database [48]). Other genomes, such as *A. niger*, *A. oryzae*, *C. immitis*, *C. posadasii*, *Ustilago maydis*, and *F. graminearum* are either already loaded or being loaded into the curation infrastructure, which is maintained in collaboration with the Wellcome Trust Sanger Institute.

**Figure 21:**
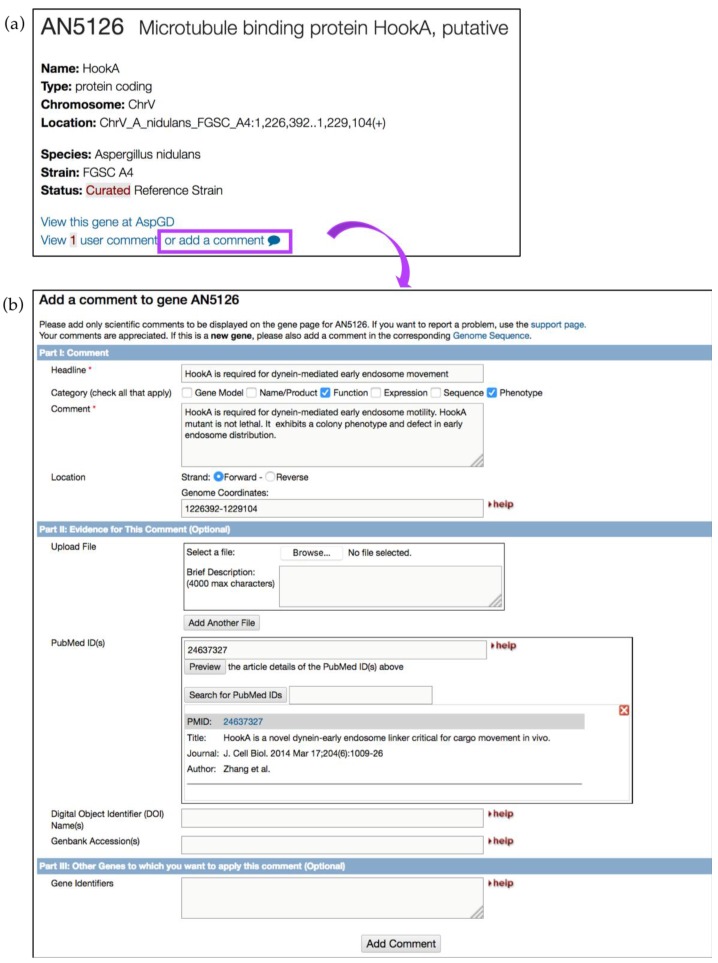
Improving gene records via user comments. (**a**) Shown is a gene record page for a putative microtubule-binding protein HookA AN5126 gene in *A. nidulans*. To add a comment, click on the *add a comment* link, which is located at the top of the gene record page (highlighted in purple); (**b**) User comment forms can be supplemented with scientific or experimental information (see comment examples in Table 2); files can be uploaded as well.

User comments offer the fastest way to improve gene records and to alert the curation team of critical literature sources. We strongly encourage the community to offer their expertise to improve gene records. User comments about new findings or publications, or even negative results, help improve genome annotations and provide evidence for alternative gene models. To add user comments, click on the *Add a comment* link available on all gene record pages (Figure 21a). Figure 21b shows a user comment form where a user can enter descriptive information about gene function, upload a reference and files (e.g., images of protein tracking), and/or other gene identifiers. Several examples of user comments are shown in Table 2. All user comments become immediately visible on the gene pages, searchable via the text search, and can be modified at any time from a user account. To upload user comments in bulk, email *help@fungidb.org.*

## 10. Conclusions

FungiDB is a service-oriented resource created with the primary goal of providing fungal researchers with access to data and with tools and functionality that enable them to ask their own questions. While it is critical that all datasets are loaded carefully, and quality controlled before public release, FungiDB strives to enable data mining to drive novel testable hypotheses rather than only recapitulating published conclusions. FungiDB benefits tremendously from being part of EuPathDB since resources can be more efficiently allocated and functionality can be broadly applied across databases. Future developments in FungiDB will include improved data mining tools, expanded workspaces for primary data analysis and integration, dedicated curatorial effort for supported genomes, and integration of new datatypes and system-wide analysis tools. FungiDB will continue to rely on the guidance of the fungal research community through advisory groups and by direct communication with members of the community at scientific meetings or through emails to *help@fungidb.org*.

## Figures and Tables

**Table 1:** Examples of special characters used in Perl-style regular expressions.

Character	Meaning
A, B, C …	Amino acid single letter abbreviation for peptide motifs
[^a]	Any character except a
{n,m}	Match the preceding character between n and m times
[ ]	Match any character contained in the bracket

**Table 2:** Examples of user comments.

Comment type	Example Comment
Gene name, gene synonym	Cell division control protein Cdc48 is also known as dsc-6
Functional characterization	This is a transcription factor involved in the regulation of copper import
Subcellular localization	GFP tagging demonstrates that ham-5 co-localize with mak-2. Images attached
Phenotype	Deletion of rim101 has resulted in capsule defects and increased virulence in mice models
